# Echocardiographic Assessment of Cardiac Function in Mouse Models of Heart Disease

**DOI:** 10.3390/ijms26135995

**Published:** 2025-06-22

**Authors:** Nadia Salerno, Assunta Di Costanzo, Fabiola Marino, Mariangela Scalise, Isabella Leo, Jolanda Sabatino, Giovanni Canino, Antonio Leccia, Antonella De Angelis, Konrad Urbanek, Daniele Torella, Eleonora Cianflone

**Affiliations:** 1 Department of Experimental and Clinical Medicine, Magna Graecia University, 88100 Catanzaro, Italy; nadia.salerno@unicz.it (N.S.); marino@unicz.it (F.M.); m.scalise@unicz.it (M.S.); isabella.leo@unicz.it (I.L.); sabatino@unicz.it (J.S.); canino@unicz.it (G.C.); 2 Department of Medical and Surgical Sciences, Magna Graecia University, 88100 Catanzaro, Italy; assuntadicostanzo.med@gmail.com; 3 Cardiology Complex Unit, ASL Napoli 2 Nord “San Giovanni di Dio” Hospital, 80027 Frattamaggiore, Italy; antonio.leccia@aslnapoli2nord.it; 4 Department of Experimental Medicine, University of Campania “Luigi Vanvitelli”, 80138 Naples, Italy; antonella.deangelis@unicampania.it; 5 Department of Molecular Medicine and Medical Biotechnologies, University of Naples “Federico II”, 80131 Naples, Italy; konradarkadiusz.urbanek@unina.it

**Keywords:** echocardiography, cardiac imaging, heart failure, small animals, mice

## Abstract

Echocardiography is a cornerstone technique for evaluating cardiac function in preclinical research using murine models. This review provides a comprehensive overview of the echocardiographic approaches employed to assess ventricular function in mouse models of heart disease, highlighting methodological principles, technical challenges, and the translational relevance of findings. Various echocardiographic modalities enable the precise evaluation of systolic and diastolic function. This article emphasizes standardization in image acquisition and analysis to minimize inter-operator variability and ensure reproducibility. It details echocardiographic parameters and strain imaging across commonly used mouse models of non-ischemic dilated cardiomyopathy, diabetic cardiomyopathy, hypertensive heart disease, and ischemic heart disease. Furthermore, it explores the advantages and limitations of anesthesia, probe positioning, and physiological monitoring during imaging. The integration of advanced imaging technologies such as Speckle-Tracking Echocardiography (STE), Three-Dimensional (3-D), and Four-Dimensional (4-D) echocardiography is discussed as a promising avenue for enhancing data quality and improving the translational potential of preclinical cardiac studies.

## 1. Introduction

Echocardiography is the most widely used diagnostic technique in cardiology, recognized as the gold standard in clinical practice for the evaluation of cardiac morphology and function due to its simplicity, reproducibility, rapidity, non-invasiveness, and low cost [1]. The application of the echocardiographic method in small animals is widely used in preclinical research and enables the evaluation of changes in cardiac structure and function in various models of heart disease [2].

The objective of preclinical research is to investigate the pathophysiology of heart disease or the response to pharmacological treatments in different heart disease models, with the goal of obtaining results that can be translated into clinical practice. Therefore, providing a detailed description of cardiac injury in animal models is critical for a comprehensive understanding of the underlying mechanisms involved in innovative therapies. This step is essential for translating the observed benefits into studies involving larger animal models and, eventually, into clinical research.

For this purpose, echocardiographic monitoring provides a wealth of information. Using dedicated equipment and software, it is possible to assess both global systolic and diastolic function, as well as regional function in rodents.

However, several limitations are associated with image acquisition and data analysis [3]. The main limitation is the high inter-observer variability, which can lead to poor reproducibility of the results. Other limitations include the lack of structured training for operators, poor standardization of measurements, limited adherence to reference guidelines, and low translation rates of preclinical findings into clinical practice.

Therefore, the aim of this review is to provide a comprehensive overview of echocardiographic techniques used in adult mice in various models of myocardial injury, with the goal of standardizing analyses and improving data quality in preclinical research.

## 2. Echocardiographic Imaging Techniques in Murine Models

### 2.1. Preparation, Anesthesia, and Imaging Techniques

To effectively perform transthoracic echocardiography (TTE) in adult mice, appropriate animal preparation and the acquisition of high-quality images are essential.

The most commonly used animals in preclinical models of cardiac injury are C57BL/6 mice [4]. This strain offers good reproducibility due to its well-characterized genetics. The standard age to define an adult mouse is approximately 8 weeks [5]. Typically, the preferred time window for preclinical studies ranges between 8 and 12 weeks. During this period, the animal’s weight is approximately 22–35 g. Body weight (BW) is essential for the calculation of indexed parameters and for the appropriate selection of the echocardiographic probe (Table 1).

The procedure can be conducted in either conscious or anesthetized mice [6]. Anesthetized mice are generally preferred, as imaging in conscious animals requires a training period, greater operator expertise, or the involvement of multiple operators. Moreover, stress-related tachycardia may also compromise data quality. However, the choice of anesthesia is a critical determinant in murine echocardiography, as it directly affects cardiac function, particularly the heart rate and ejection fraction. Intraperitoneal anesthesia—using substances like ketamine/xylazine, tribromoethanol, barbiturates, or diazepam—tends to decrease the heart rate and ventricular contractility, exerting negative chronotropic and inotropic effects. In contrast, inhalation anesthesia—with agents like isoflurane, sevoflurane, or halothane—exerts minimal impact on cardiac function, enabling more accurate and reproducible measurements; therefore, it is preferred [8,9]. Isoflurane (1–2%) is most commonly used due to its titratability and relatively mild cardiovascular depression, allowing heart rates to remain within a physiological range (>400 bpm). In contrast, ketamine–xylazine, although once widely used, has been shown to cause significant bradycardia and suppression of left ventricular function in a dose-dependent manner, largely due to the xylazine’s α2-adrenergic effects. As shown by Pachon et al., isoflurane yields significantly higher ejection fractions compared to ketamine–xylazine or pentobarbital, reinforcing its utility in functional imaging [10]. Furthermore, accurate assessment of diastolic function also requires careful control of heart rate, as rates > 600 bpm can lead to fusion of the E and A filling waves, making analysis difficult. Ultimately, the choice of anesthetic should balance ease of use, physiological stability, and the specific functional parameters being measured. Prior to image acquisition, the precordial region should be shaved, and acoustic coupling gel at an appropriate temperature should be applied to minimize acoustic impedance. The animal should be placed on a heated platform to prevent hypothermia during the procedure, and electrocardiographic (ECG) monitoring should be performed using electrodes attached to the limbs [11].

Technological advances have facilitated the development of imaging systems capable of acquiring high-resolution images suitable for small animal models [12]. In murine echocardiography, high-frequency linear array transducers are employed to minimize imaging artifacts. These probes typically operate within a frequency range of 30–50 MHz (up to 70 MHz), offering an axial resolution down to 50 µm and imaging depths of 5–12 mm. In general, higher probe frequencies are selected for animals with lower body mass. Specifically, a 30 MHz probe is recommended for BW > 35 g, while a 40 MHz probe is more appropriate for BW < 35 g, ensuring a real-time imaging frame rate of >30 frames per heartbeat [13].

The standard examination is usually performed with a frame rate > 100 frames per second and an average imaging depth of 11 mm. The frame rate should be increased in cases of elevated heart rate and for advanced analyses, such as speckle tracking analysis [14,15]. For Doppler studies, the probe must be capable of sampling peak velocity values greater than 1500 mm/s [6]. In conscious adult mice, heart rate typically ranges between 600 and 700 beats per minute (bpm); in anesthetized mice, image acquisition should ideally occur at a heart rate of ≥400 bpm and optimally ≥ 450 bpm [6].

### 2.2. Standard Views and Functional Modalities

The echocardiographic transducer is positioned at specific anatomical landmarks on the animal’s thorax to acquire standardized imaging planes, referred to as echocardiographic windows [16] (Table 2).

**Figure 1:**
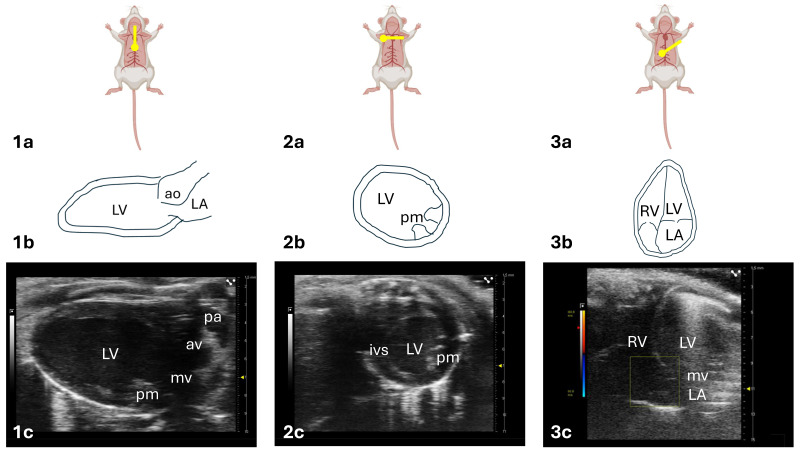
Standard echocardiographic views in mice. This figure illustrates the main echocardiographic views used to assess cardiac function in mice through TTE. The image is organized into three columns (1, 2, and 3), each corresponding to a different echocardiographic window. (1) Parasternal Long-Axis View (PLAX): the transducer should be parallel to the central axis of mouse body and orientated at the lower one-third of the chest with the indicator marker toward the left hip (1a); the left ventricle (LV) is visualized longitudinally along the probe’s axis, with the apex oriented towards the left side, the mitral valve (mv) is visible at the lower portion of the image, while the aortic valve (av) and the aortic root appear at the top (1b); the ultrasound image shows a PLAX view, enabling assessment of ventricular size and function (1c). (2) Parasternal Short-Axis View (PSAX): the PSAX view is obtained by rotating the transducer 90 clockwise from the PLAX view, with the indicator marker toward the right shoulder (2a); a cross-sectional view of the left ventricle (2b); the ultrasound image shows a PSAX view, enabling evaluation of ventricular wall thickness, chamber dimensions, and myocardial contractility (2c). (3) Apical 4-chamber View (A4C): the transducer should be moved above the left side of the xiphoid, rotated counterclockwise by about 45°, and tilted by about 30–60° relative to the platform (3a); left ventricle (LV) and right ventricle (RV) appear in the upper portion, separated by the interventricular septum (ivs); left atrium (LA) and right atrium (RA) are visualized in the lower portion, separated by the interatrial septum (3b); the ultrasound image shows a A4C view, commonly used in mouse cardiac imaging to simultaneously visualize all four cardiac chambers, enabling comprehensive assessment of chamber sizes, cardiac function, ventricular and atrial volumes, and valvular function, as well as detection of abnormal flow patterns (3c). Table Note: ao: aorta; av: aortic valve; ivs: interventricular septum; LA: left atrium; LV: left ventricle; mv: mitral valve; pa: pulmonary artery; pm: papillary muscle; RV: right ventricle.

The three primary windows employed in murine echocardiography are the parasternal long-axis (PLAX) view, the parasternal short-axis (PSAX) view, and the apical four-chamber (A4C) view (Figure 1).

Parasternal views are acquired with the animal placed in a supine position on a horizontal, temperature-controlled platform. The echocardiographic probe is positioned on the left side of the thorax.

Regarding the PLAX view (Figure 1(1a)), the probe notch (or marker) should be oriented toward the left hip of the mouse. In this orientation, the left ventricle (LV) appears longitudinally aligned with the probe, with the apex directed to the left, the mitral valve located inferiorly, and the aortic valve and aortic root located superiorly.

A 90° clockwise rotation of the probe from the PLAX view yields the PSAX view (Figure 1(2a)), with the probe marker now directed toward the animal’s right shoulder. In this view, the right ventricle (typically crescent-shaped) and the LV (normally circular) are visualized in the cross-section. If the LV appears oval rather than round, the imaging plane is likely oblique rather than perpendicular to the ventricular axis. By angling the probe cranially to caudally, it is possible to obtain sequential cross-sectional images of the basal, mid-ventricular, and apical segments of the left ventricle.

The A4C view (Figure 1(3a)) is obtained by tilting the heating platform laterally to the left and positioning the animal in a Trendelenburg-like orientation, with a 25–30° incline so that the head and thorax are positioned lower than the hind limbs. The probe is placed at the apical region of the chest, with the marker directed toward the animal’s left flank. In this view, the left cardiac chambers appear on the right side of the screen (LA inferiorly, LV superiorly), separated by the mitral valve, while the right cardiac chambers appear on the left side (right atrium inferiorly and right ventricle superiorly), separated by the tricuspid valve.

The main echocardiography modalities applied to imaging in rodents include the B-mode (two-dimensional brightness mode or 2-D echo), M-mode (motion mode), color Doppler or color flow mapping (CFM), Speckle-tracking echocardiography (STE), and three-dimensional (3-D echo) and four-dimensional (4-D echo) imaging [15].

The B-mode is the classic two-dimensional black-and-white image representation and is applied to all echocardiographic views. In these images, hyperechoic structures appear bright (white), while hypoechoic regions, such as blood-filled chambers, appear dark (black).

The heart undergoes continuous periodic movements—systole and diastole—and it is often useful to visualize these dynamic phases along a fixed scan line. M-mode echocardiography provides a temporal representation of the motion of individual anatomical structures along a single line of interrogation. Unlike B-mode imaging, where successive echoes overlap spatially to create a two-dimensional image, M-mode imaging displays echoes side by side over time, allowing for the precise assessment of motion and structural changes along the scan line.

To contextualize the anatomical origin of the M-mode trace, a real-time B-mode image is typically displayed at the top of the monitor, with a dotted line indicating the scan line used for M-mode acquisition. M-mode imaging can be applied in both long-axis and short-axis views to evaluate left ventricular dimensions during systole and diastole periods, as well as to assess systolic function.

### 2.3. Assessment of Left Ventricular Systolic Function

**Figure 2:**
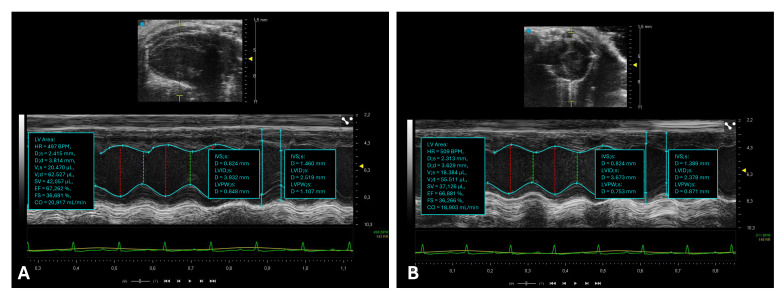
Assessment of left ventricular systolic function in mice. This figure illustrates echocardiographic assessments of left ventricular systolic function in mice using M-mode ultrasound imaging. (**A**) M-mode echocardiogram with measurements of left ventricular dimensions during systole and diastole, acquired from a parasternal long-axis (PLAX) view. (**B**) M-mode echocardiogram with measurements of left ventricular dimensions during systole and diastole acquired with a PSAX view.

Systole represents the phase of myocardial contraction during which blood is ejected from the cardiac chambers into the circulation [21]. Key parameters used to quantify systolic function include the ejection fraction (EF) and fractional shortening (FS) (Figure 2) [22,23,24].

EF is the most widely used echocardiographic index for assessing systolic function. It represents the percentage of blood ejected from the LV during systole. EF is calculated as the ratio of stroke volume, the difference between the left ventricular end-diastolic volume (LVEDV), and the left ventricular end-systolic volume (LVESV) divided by LVEDV and multiplied by 100:

$$EF=\frac{LVEDV-LVESV}{LVEDV}*100$$

FS measures the degree of myocardial fiber contraction along the longitudinal axis of the left ventricle. FS is measured as the percentage reduction in the left ventricular internal diameter from diastole to systole. Specifically, FS is determined by the difference between left ventricular end-diastolic internal diameter (LVIDd) and left ventricular end-systolic internal diameter (LVIDs) divided by LVIDd and multiplied by 100:

$$FS=\frac{LVIDd-LVIDs}{LVIDd}*100$$

The LVIDd and LVIDs measurements of the LV are performed using M-mode echocardiography, in the PLAX (Figure 2A) and PSAX (Figure 2B) view, at the level of the papillary muscles.

The image is first acquired in the B-mode to correctly identify the section, then the M-mode is activated by placing the cursor so that it crosses the center of the left ventricle, perpendicular to the anterior and posterior walls.

End-diastole is the moment when the LV is most dilated, just before contraction, and corresponds to the peak of the R wave on the ECG. LVIDd is measured from the anterior endocardium to the posterior endocardium at the end of diastole. End-systole is the moment of maximum contraction of the ventricle, when it has the smallest diameter, at the end of systole. LVIDs is measured from the anterior endocardium to the posterior endocardium at the end of systole. The measurements are expressed in millimeters (mm).

By evaluating the diameters, it is possible to determine the values of LVEDV and LVESV using the Teichholz formula:

$$V=\left(\frac{7}{2.4+D}\right)*D^{3}$$

where V is the volume, and D is the diameter. This method assumes an ellipsoidal geometry of the ventricle, which may not be accurate in the presence of structural abnormalities. It is less precise than Simpson’s method, but it is simpler and quicker to perform [25].

Small abnormalities in wall motion may not be detected through B-mode or M-mode assessment.

The Simpson’s method, adapted for echocardiography in mice, is an effective tool for assessing left ventricular function through ventricular volumes, particularly in murine models presenting structural abnormalities, such as myocardial infarction (MI) models [6]. This method involves tracing the endocardial borders of the LV during systole and diastole to obtain the area of the ventricular cavity in each section. By multiplying the traced area of each section by the section thickness, the volume of each segment (disk) is calculated. The total left ventricular volume is then derived by summing the volumes of all individual sections (the disk summation method) [16].

3-D echo has proven effective in measuring left ventricular volumes and in evaluating both global and regional cardiac function in murine models [16]. In particular, it is possible to obtain the stroke volume (SV = End-diastolic volume − End-systolic volume), cardiac output (CO = SV × Heart Rate), and left ventricular mass (LVM = Epicardial Volume − Endocardial Volume × 1.06 g/µL, where 1.06 g/µL represents the specific gravity of myocardial tissue) [26]. The 3-D echo technique in mice is based on geometric reconstruction of the mouse LV from multiple 2-D images [27,28]. The two most common approaches are the biplane method, which combines the left ventricular length from the PLAX view with three PSAX sections to reconstruct the ventricular volume, and the sequential PSAX method, in which PSAX images are acquired at regular intervals (approximately 1 mm) along the long axis of the left ventricle, allowing for a more detailed volumetric reconstruction. A study demonstrated that the sequential PSAX method provides a more accurate estimation of ventricular volumes and EF compared to the standard PLAX-based method, showing strong agreement with cardiac magnetic resonance (CMR) imaging, which is considered the gold standard [29].

More recently, a tool for 4-D echo assessment of cardiac function has been introduced [30]. 4-D echo data are obtained using an ultrasound probe mounted on a linear motor that moves it along the short axis of the LV [31]. The system automatically acquires high-frequency cine loops synchronized with both the cardiac and respiratory cycles. The acquired images are compiled in space and time to create a 4-D echo dataset representing the heart in motion throughout the cardiac cycle. By manually tracing the endocardial and epicardial contours, a volumetric mask of the LV is created, allowing for the evaluation of parameters such as LVEDV, LVESV, EF, and LVM [32].

### 2.4. Assessment of Left Ventricular Diastolic Function

The A4C view enables comprehensive visualization of all cardiac chambers and is particularly well-suited for Doppler analysis [33]. By adjusting the transducer orientation, transvalvular flows can be aligned for accurate velocity assessment. Doppler techniques—including color flow (CFM), continuous-wave (CWD), pulsed-wave (PWD), and tissue Doppler imaging (TDI)—are used to evaluate flow and myocardial motion. Color Doppler depicts flow direction and turbulence, while CWD and PWD offer velocity profiles: CWD for high-velocity flows without depth specificity, and PWD for site-specific measurements with a limited velocity range due to aliasing. These modalities are commonly used to assess blood flow across the atrioventricular valves (mitral and tricuspid) and the aortic valve.

**Figure 3:**
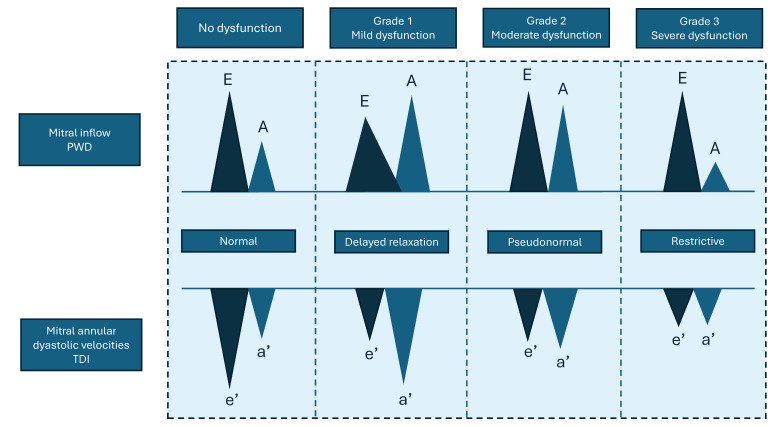
Patterns of left ventricular diastolic dysfunction assessed using mitral inflow and tissue Doppler imaging. This image illustrates the progression of left ventricular diastolic dysfunction from normal function to severe impairment (Grade 3). It compares mitral inflow patterns assessed using pulsed-wave Doppler (PWD) with mitral annular diastolic velocities measured using tissue Doppler imaging (TDI). Key features include changes in the E/A ratio and e′/a′ velocities, helping to distinguish between normal relaxation, delayed relaxation, pseudonormal, and restrictive filling patterns.

The assessment of diastolic function is a complex field, and the applicability of echocardiographic parameters commonly used in humans has not yet been extensively validated in various murine pathophysiological models (Figure 3) [34].

The main determinants of diastolic function are left ventricular relaxation during early diastole (or protodiastole) and left ventricular compliance during mid- to late diastole (or meso-telediastole). There are four phases of diastolic function: isovolumic release; rapid filling (associated with the E wave or E), during which approximately 80% of ventricular filling occurs; diastasis; and atrial contraction (associated with the A wave or A), which contributes an additional 15% to ventricular filling.

**Figure 4:**
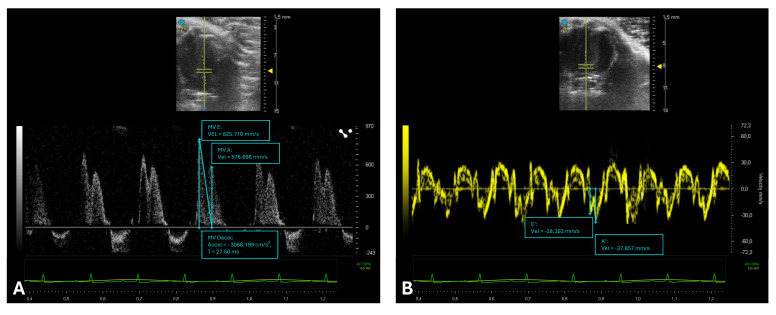
Assessment of diastolic function in mice. This figure illustrates echocardiographic assessments of diastolic function in mice obtained from an apical four-chamber (A4C) view using pulsed-wave Doppler (PWD) and tissue Doppler imaging (TDI). (**A**) PWD capture transmitral flow velocities: early diastolic ventricular filling (E-wave), late diastolic ventricular filling due to atrial contribute (A-wave), and E-wave deceleration time (DT), reflecting diastolic relaxation and ventricular filling dynamics (diastasis). (**B**) TDI assesses myocardial tissue velocities at the mitral annulus level: early diastolic myocardial velocity (e′ wave) and late diastolic myocardial velocity (a′ wave), critical parameters for assessing myocardial relaxation properties and ventricular diastolic function.

PWD of transmitral inflow allows for the measurement of E and A waves, their ratio (E/A), and the deceleration time (DT), reflecting LV relaxation and compliance. Impaired relaxation results in a reduced E wave and prolonged DT, whereas increased stiffness leads to steep E-wave deceleration and decreased A wave due to elevated filling pressures. Mixed diastolic dysfunction can show both patterns. Yang and colleagues were the first to investigate the effectiveness of transmitral Doppler echocardiography in assessing left ventricular diastolic function in mice [35]. To measure these parameters, an image of the mitral valve must be obtained in the A4C view (Figure 4).

The transducer should be positioned above the left side of the xiphoid, perpendicular to the central axis of the mouse’s body, rotated counterclockwise by approximately 45°, with a tilt of 30–60° relative to the platform. The Doppler spectrum of the mitral inflow can be easily recorded using the PWD mode. The sample volume is placed at the level of the mitral valve orifice, just beyond the valve leaflets, to measure the inflow into the LV during diastole, which displays an E (early diastolic ventricular filling) and an A (late diastolic filling due to atrial contraction) (Figure 4A) [36]. In addition to traditional Doppler indices such as mitral inflow velocities, parameters like the pulmonary venous blood flow waveform and left ventricular inflow propagation velocity obtained via the Color M mode have been demonstrated to provide complementary and valuable information on murine diastolic function. The pulmonary venous flow pattern reflects left atrial pressure and ventricular filling dynamics, offering insights that can help differentiate between normal and pathological diastolic states [35]. Similarly, the inflow propagation velocity is considered a less load-dependent marker of myocardial relaxation and has been shown to improve the accuracy of diastolic function assessment in mice [37,38]. However, it is important to note that these measurements require high-resolution echocardiographic equipment and technical expertise, given the small size and rapid heart rate of murine models. Consequently, their application may be limited in some settings. Nonetheless, incorporating these parameters into echocardiographic protocols enhances the comprehensiveness of diastolic function evaluation and is encouraged in studies investigating subtle or early cardiac dysfunction.

A more recent advancement in the assessment of diastolic function is TDI, a technique that allows for the direct analysis of myocardial velocities at the tissue level, as described well in a study by Schnelle and colleagues [20,39]. TDI is typically acquired at the lateral mitral annulus and provides insights into longitudinal myocardial motion. The resulting waveform typically displays two positive peaks during systole and two negative peaks during diastole: an early diastolic peak (e′) and a late diastolic peak (a′) (Figure 4B). The e′ peak is considered a robust index of myocardial relaxation. A reduced e′ suggests impaired ventricular relaxation and reduced early diastolic suction. Conversely, e′ may increase in states of elevated preload [40]. Importantly, the E/e′ ratio—obtained by dividing the transmitral E velocity by TDI-derived e′—is widely used to estimate LV filling pressures [41]. A high E/e′ ratio indicates elevated LA pressure and is associated with diastolic dysfunction [42].

LA size is typically assessed in PLAX or A4C views by tracing the endocardial border of the LA [43,44]. The measurement should be performed at end-systole, just prior to mitral valve opening, when the LA volume is at its maximum [45]. An increased LA area is a surrogate marker of chronically elevated left ventricular filling pressures and may indicate underlying diastolic dysfunction [46].

Another important parameter for assessing diastolic function is the isovolumic relaxation time (IVRT) [19]. IVRT is measured using Doppler imaging in the A4C view by simultaneously recording mitral inflow and aortic outflow profiles. This allows for the measurement of the time interval between aortic valve closure and mitral valve opening.

When LV relaxation is impaired, the decline in ventricular pressure is slower, delaying mitral valve opening and prolonging IVRT. A prolonged IVRT is indicative of impaired relaxation, while a shortened IVRT is characteristic of restrictive diastolic filling patterns, typically associated with elevated LA pressure.

### 2.5. Speckle-Tracking Echocardiography

After image acquisition, post-processing is performed using specialized software, where speckle-tracking echocardiography (STE) can be applied to quantitatively assess myocardial deformation independently of the ultrasound beam angle. STE tracks natural acoustic reflections, or “speckles”, within the myocardium frame-by-frame throughout the cardiac cycle, enabling the evaluation of key parameters: displacement (speckle movement), velocity (speed of displacement), strain (degree of myocardial deformation), and strain rate (speed of deformation).

Moreover, to evaluate regional function and mechanical desynchrony, Time-to-Peak (TTP) analysis was performed across six myocardial segments, providing insights into segmental strain and strain rate patterns.

**Figure 5:**
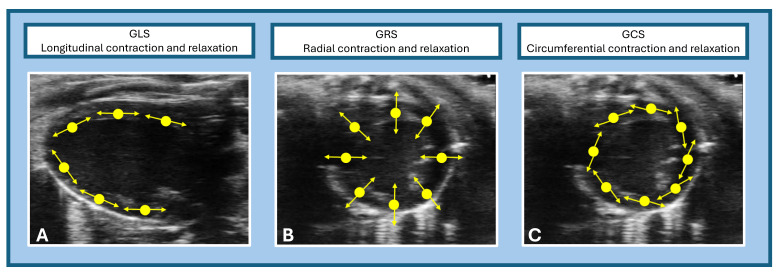
Multidimensional assessment of myocardial strain using speckle-tracking echocardiography (STE). This image illustrates the three principal components of myocardial deformation assessed by STE. (**A**) GLS (global longitudinal strain) evaluates myocardial contraction and relaxation along the long axis of the heart, reflecting subendocardial fiber function. (**B**) GRS (global radial strain) measures myocardial thickening and thinning in the radial direction from the endocardium to the epicardium. (**C**) GCS (global circumferential strain) assesses myocardial shortening along the circular axis of the ventricle.

STE assesses myocardial deformation in three principal directions (Figure 5):

Global longitudinal strain (GLS, Figure 5A) measures contraction along the long axis of the left ventricle (LV), typically from the parasternal long-axis (PLAX) view, with normal values around −22%;
Global radial strain (GRS, Figure 5B) reflects myocardial thickening and thinning perpendicular to the wall, assessed from the parasternal short-axis (PSAX) view, with normal values near +35%;
Global circumferential strain (GCS, Figure 5C) represents circumferential shortening around the LV and from PSAX, with normal values around −30%.

Regional function and mechanical synchrony were further evaluated by Time-to-Peak (TTP) analysis across multiple myocardial segments, revealing segmental strain patterns and potential desynchrony.

High-quality 2D images, synchronized with ECG, are essential for accurate strain assessment. Operators define the region of interest by marking the LV endocardial border, allowing the software to track speckles across the myocardium and generate quantitative strain curves and visual maps. This semi-automated approach facilitates the verification of tracking accuracy.

**Figure 6:**
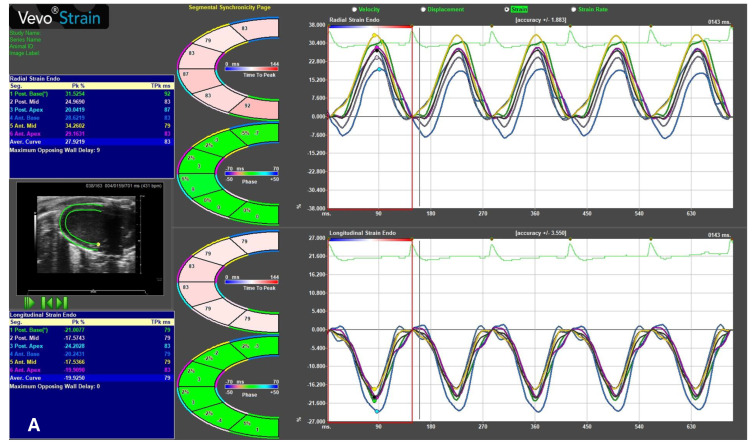

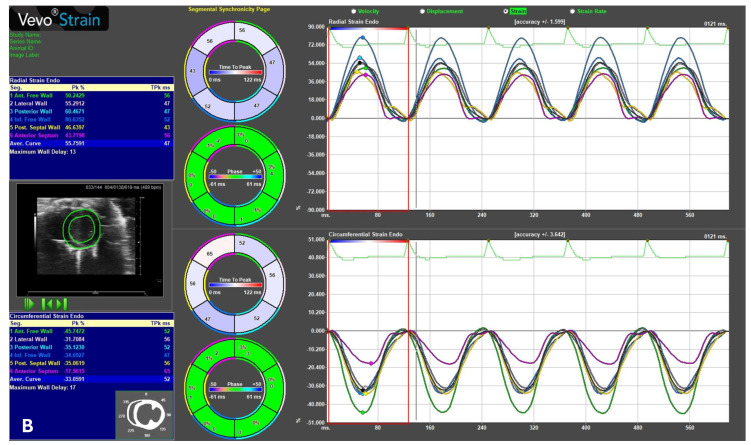
Speckle-tracking echocardiography (STE) in mice. Advanced cardiac analysis (regional and global cardiac measurements) assessed using STE (Vevo Strain 2.0, Vevo LAB analysis software; VisualSonics, Toronto, ON, Canada). (**A**) Global longitudinal strain (GLS), measured from a PLAX view of the left ventricle, indicates myocardial shortening during systole, represented by negative percentage values (normal reference values around −22%). (**B**) Global radial strain (GRS) and global circumferential strain (GCS), obtained from the PSAX view; GRS, reflecting myocardial thickening during systole, is expressed in positive percentage values (normal reference values around +35%); GCS, indicative of myocardial circumference shortening during systole, is expressed as negative percentage values (normal reference values around −30%).

STE provides a sensitive and multidimensional evaluation of myocardial function, often detecting early dysfunction before changes in conventional parameters like the ejection fraction. Recent studies have extended STE to assess left atrial function in mice, offering new insights into atrial pathophysiology (Figure 6) [17].

## 3. Echocardiography in Murine Models of Heart Diseases

### 3.1. Echocardiography in Murine Non-Ischemic Dilated Cardiomyopathy

Dilated cardiomyopathy (DCM) is a myocardial pathology characterized by left or biventricular dilatation and impaired contraction with subsequent systolic and diastolic dysfunction, not secondary to ischaemic, valvular, or congenital heart disease [47].

The incidence of DCM in humans is increasing, and its true prevalence is still underestimated [48,49]. The aetiology is heterogeneous, associated in 40% of cases with genetic causes and in the remaining cases with nongenetic forms (infection, toxic, drug, autoimmune, or metabolic diseases). The treatment of DCM has evolved over the years, but unresolved issues remain in the management of non-responder patients and asymptomatic individuals.

Murine models have proven useful for studying the development and progression of the non-ischemic dilated phenotype, and several models are available for testing new therapeutic strategies (Table 3).

Cardiotoxicity mediated by antineoplastic drugs is a major problem in cancer patients because of the development of DCM, onset of arrhythmias, reduced EF, and congestive heart failure, which can lead to the death of the patient [59]. The cardiotoxic action of doxorubicin is due to a dual mechanism of action: the first mechanism is the formation of reactive oxygen species, and the second mechanism is mitochondrial damage due to the formation of the doxorubicin-topoisomerase 2β complex that can bind and break the DNA of cardiomyocytes and cause their cell death [50]. The administration of doxorubicin to animals enabled the development of a mouse model of non-ischemic DCM [32].

Doxorubicin is administered to mice intraperitoneally. In the acute model, mice receive a single injection of high-dose doxorubicin (15–20 mg/kg). After a single dose, there is an initial increase in left ventricular diameter at 3 days and a marked reduction in EF and FS at 5 days, with high mortality. Systolic dysfunction is due to an elevated inflammatory response, cardiomyocyte death by apoptosis, and fibrosis in cardiac tissues [60]. In the chronic model, administration includes lower (4–5 mg/kg) and repetitive doses of doxorubicin. After 12 weeks from the start of treatment with chronic doses, there is a reduction in left ventricular thickness and mass, an increase in ventricular diameter, and a reduction in EF and FS [61,62,63,64]. In addition, doxorubicin treatment has been associated with a significant reduction in diastolic indices, particularly the ratio of the mitral inflow velocity to early mitral annular diastolic velocity (E/E′). Currently, there is no cardioprotective drug that can counteract the effects of antineoplastics and is free of side effects [65,66]. Therefore, this model has been widely used to evaluate the effectiveness of cardiac function-enhancing strategies in doxorubicin-induced toxic damage to cardiomyocytes [51,62].

Another pattern of toxic injury was recently described following subcutaneous administration of high-dose (400 mg/kg) Isoproterenol (ISO), a β-adrenergic agonist that induces cardiac stress [67,68]. ISO is a sympathomimetic drug active on betadrenergic receptors. Several in vivo and in vitro studies have shown that activation of beta-2 receptors activates the signal transduction pathway, resulting in hypertrophy at low levels of expression and apoptosis of cardiomyocytes at high levels of regulation [69]. In fact, mice treated with a high dose of ISO (400 mg/kg) develop stress-induced cardiomyopathy (SIC) with cardiac hypertrophy, cardiomyocyte necrosis, fibrosis, increased LV end-systolic diameter, diastolic dysfunction, and HF, as found in ischemic cardiomyopathy [70]. This model simulates Takotsubo syndrome, a syndrome with high mortality and morbidity, characterized by akinesia of apical segments with normal function of basal segments [68]. Severe segmental and global cardiac dysfunction occurs within two hours of administration, but this impairment gradually resolves, and both systolic and diastolic function return to baseline levels by 14 and 28 days post-injection. No significant myocardial fibrosis was observed at that time [52,71].

In contrast, mice that received continuous 5-fluorouracil (5-FU) treatment after ISO administration, starting at 14 days and continuing through 28 days, exhibited a progressive increase in LV end-systolic and end-diastolic diameters, resulting in EF and FS depression. Moreover, a significant increase in E/e′ was observed as an index of diastolic dysfunction, all typical features of dilated cardiomyopathy. Assessment of myocardial deformation revealed that ISO significantly reduced GLS, which normalized by days 14 and 28. Conversely, the administration of 5-FU after ISO caused sustained worsening of GLS at 14, 28, and 56 days (−12.44 ± 3.55 vs. −19.62% ± 2.56) [53]. In contrast, the chronic administration of ISO more closely mimics a model of advanced HF [72].

Based on the evidence related to the occurrence of cardiomyopathy from chronic alcohol abuse in humans, a mouse model of alcoholic cardiomyopathy was tested: chronic ethanol consumption (4%) resulted in the onset of cardiac dysfunction after 8 weeks in mice. Specifically, the animals developed non-ischemic DCM with increased diameters and decreased EF and FS in response to chronic exposure to alcohol [54].

Homocysteine, in addition to diet, also allows a model of DCM to be reproduced. The GENICA study demonstrated an inverse association between plasma homocysteine levels and left ventricular systolic function, describing increased cardiovascular mortality in subjects with hyperhomocysteinemia [55]. Based on this study, the murine model was developed: mice fed a homocysteine-enriched diet developed increased left ventricular size, reduced FS, and increased PR and QRS interval at approximately 12 weeks after treatment initiation [73].

Mice with a genetically induced dilated phenotype are widely used for DCM protocols [74,75]. The tropomyosin 230-point mutation (D230NcTm) causes reduced Ca^2+^ sensitivity of troponin C in the sarcomere, resulting in reduced cardiac contractility. D230NcTm mice developed DCM between two and five months of age with progressive and significant systolic dysfunction and ventricular dilatation [56]. A more severe dilated phenotype with reduced lifespan has been described for troponin C mutations (I61Q and D73N), which cause desensitization to Ca^2+^. Such mutations accelerate the dissociation of Ca+ from cTnC, which results in reduced contractile force and thus the development of DCM [57].

Mice with the D73N (D73NcTnC) and I61Q (I61QcTnC) mutation developed early-onset DCM, with substantially reduced EF, increased left ventricular size, reduced wall thickness, and sudden death, mainly from arrhythmic causes (QT and QRS prolongation) [57,58]. The median survival was about 12 weeks.

### 3.2. Echocardiography in Murine Diabetic Cardiomyopathy

The incidence of diabetes mellitus is increasing in the general population and is closely linked to the development of diabetic cardiomyopathy, with a history of heart failure being twice as likely in men with diabetes and five times more likely in women with diabetes [76]. The pathogenesis of diabetic cardiomyopathy is complex and multifactorial, and cardiac damage is mainly associated with increased oxidative stress, altered mitochondrial function, increased fatty acids and blood glucose, cardiac remodeling, and cellular apoptosis (inflammation, fibrosis, and necrosis) [77,78]. Diabetic cardiomyopathy can manifest as either heart failure with a preserved ejection fraction (HFpEF) or heart failure with a reduced ejection fraction (HFrEF) [79]. In the early stages, it often presents with diastolic dysfunction and preserved systolic function, corresponding to HFpEF. However, as the disease progresses, systolic impairment may develop, leading to a reduced EF and a transition toward HFrEF.

Murine models of DCM are also widely used to describe the cardiac response to metabolic disorders associated with diabetes mellitus (Table 4) [80]. Major mouse models of type 1 diabetes mellitus (T1D) and type 2 diabetes mellitus (T2D) have been reproduced through genetic manipulation, high-fat feeding, a surgical approach, chemical or virally induced methods, or the use of pancreatic toxins [81].

The main genetic models of diabetic mice with T1D are the non-obese diabetic (NOD) mouse and the AKITA mice [89,90,91]. NOD mice are a genetic model of diabetic mice first developed in a laboratory in Osaka, Japan [82]. NOD mice, through a spontaneous autoimmune process, exhibit an initial prediabetic state in which pancreatic B cells are infiltrated by T cells (CD4 and CD8), NK cells, and B cells. In 3–4 weeks, an inflammatory process of the pancreatic islets, called insulitis, occurs. In the next 10–14 weeks, when 90% of beta cells are destroyed, the mice develop T1D. AKITA mice are C57BL/6NSlc mice that have a spontaneous mutation in the insulin 2 gene (Ins2/Cys96Tyr) [92]; under normal conditions, the gene allows for proper proinsulin folding. AKITA mice exhibit misfolding of proinsulin and the accumulation of misfolded proteins in the rough endoplasmic reticulum [93]. This leads in 3–4 weeks to the development of T1D. These two patterns are associated with hyperglycemia, hypoinsulinemia, polyuria, and polydipsia.

Echocardiographic assessments identify both AKITA and NOD mice as relevant models of HFpEF, showing preserved systolic function, normal values of EF and FS values, and normal LV diameters and wall thickness. However, these models reveal early diastolic dysfunction, evidenced by decreased velocities of E and e′ waves and an elevated E/e′ ratio [83].

Other models of T1D include chemically induced diabetes models associated with the use of alloxane and streptozotocin (STZ). These chemicals can enter pancreatic β cells through the GLUT2 transporter and impair normal pancreatic function: the inhibition of insulin secretion, necrosis of β cells, and development of insulin-dependent diabetes mellitus [85]. Alloxane (2,4,5,6 tetraoxypyrimidine; 5,6-dioxyuracil) is a chemical toxicant that enters β cells and forms free radicals through a redox cycle: alloxane is reduced to dialuric acid and subsequently reoxidized to create reactive oxygen products capable of fragmenting β cell DNA [94]. The administration of 50–200 mg/kg of alloxan in mice results in destruction of β-cells. STZ is an antifungal agent synthesized from Streptomyces Achromogenes [95]. Due to its similarity to glucose, it enters β-cells through the GLUT2 transporter. In the cell, it causes DNA alkylation, PARP activation, NAD and ATP depletion, and increased free radicals. The damage results in DNA fragmentation and cell death with subsequent inhibition of insulin production.

The most widely used model because it is practical and easy to reproduce is STZ-induced diabetes. STZ makes it possible to reproduce both T1D and T2D, which represent HFpEF and HFpEF models, respectively [84]. T1D is induced by high-dose injection of STZ (100–200 mg/kg), which causes rapid and total pancreatic beta cell failure. Eight weeks after injection; this results in a mouse model of T1D with HFrEF: increased left ventricular size (increase LVIDs/d) and systolic disfunction and impaired diastolic function.

T2D in mice is created by an initial high-fat diet (HFD) for four weeks, followed by low-dose STZ injections (20–40 mg/kg/die for 5 days) and the HFD therafter. Eight weeks later, a murine HFpEF phenotype is observed, characterized by a preserved systolic function and left ventricular diastolic dysfunction: e′ reduction, e′/a′ and E/A reduction, E/e′ increase, and GLS reduction can be observed due to reduced myocardial performance in mice [84,96,97]. In addition, Chandramouli et al. conducted a study on mice with STZ-induced diabetes and described the increased susceptibility of females to diastolic dysfunction. Specifically, the study described an early alteration in diastolic function with increased E/e′ ratio and decreased e′/a′ ratio in female mice, but not in male mice [98]. This finding appears to correlate with the higher incidence of cardiovascular mortality in diabetic females [99,100].

Regarding the diet-induced model of T2D, HFD based on lipids, sugars, or both are used to induce hyperglycemia, hyperinsulinemia, insulin resistance, and cardiac remodeling after about 8 months of exposure. Specifically, the classic chow consisting of 26% protein, 63% carbohydrate, and 11% fat has been modified with a diet rich in energy derived from fat (about 58%) [86]. In these fat-fed animals, which develop obesity and T2D, there is an increase in left ventricular thicknesses and diameter, altered diastolic function, and preserved systolic function.

Another genetically induced T2D model is the T2M model in obese mice created by leptin receptor mutation or leptin mutation. Leptin is the hormone that induces satiety. Disruption of leptin signaling occurs in these mice, leading to hyperphagia and obesity. Lepob/ob mice are leptin-deficient mice that develop weight gain and hypersinsulinemia in 2 weeks and hyperglycemia and hyperlipidemia in the following 4 weeks [87]. This model is rarely used because the animals have an increased pancreatic islet volume with little residual insulin secretion; therefore, they poorly represent the model of T2D. Hutchinson et al. demonstrated the role of increased fibroblasts and extracellular matrix in contributing to ventricular stiffness in the genetically induced mouse model of T2D (Lepob/ob mice) [101]. In this study, diabetic mice exhibited HFpEF, characterized by diastolic dysfunction without impairment of systolic function. Lepr db/db mice have an autosomal recessive mutation in the leptin receptor with nonfunctioning and insensitive leptin receptors, resulting in the development of hyperglycemia, hyperinsulinemia, obesity, and insulin resistance in 4–8 weeks [88]. These mice develop T2D with ventricular hypertrophy and HFpEF. A study on a genetic model of T2D (Leprdb/db mice) described a preserved EF with reduced cardiac contractility, ventricular release, and compliance [102]. This study highlights that, as age advances and the disease progresses, HF may evolve from a form with preserved EF to one with reduced EF [96].

### 3.3. Echocardiographic Assessment in Pressure-Overload Heart Disease Murine Models

The main causes of pressure overload in humans include arterial hypertension, aortic stenosis, aortic coarctation, and chronic pulmonary hypertension. These conditions progressively lead to concentric left ventricular hypertrophy, followed by the development of diastolic dysfunction and, subsequently, systolic dysfunction. The most common causes are undoubtedly arterial hypertension—which affects 34% of the male population and 32% of the female population—and aortic stenosis, which is the most frequent valvular heart disease, affecting 0.4% of the general population [103].

Broadly used mouse models demonstrate severe left ventricular remodeling in response to systemic arterial hypertension or aortic stenosis (Table 5) [104,105,106]. Pressure overload results in uniform left ventricular remodeling with initial ventricular wall thickening and volume reduction. The M-mode can be used to assess thicknesses and diameters (concentric hypertrophy). Subsequently, left ventricular dilatation and increased left ventricular volume are determined, with changes in EF, FS, and heart failure.

In 1991, Rockman et al. developed the first in vivo model of hypertrophic cardiomyopathy, which was used to investigate the pathophysiology of ventricular hypertrophy and HF associated with arterial hypertension and aortic stenosis [107]. Transverse aortic constriction (TAC) in mice is a minimally invasive surgical technique performed via partial thoracotomy and aortic suturing with silk thread to determine the pressure overload [108]; banding can be performed at different levels of the aorta (ascending tract, aortic arch, or descending aorta), with phenotypic variability dependent on the degree of constriction achieved by the ligation [109,110,111].

An abdominal bandage allows adaptation over time to peripheral resistance with the development of cardiac hypertrophy within 2 weeks because the proximal portion of the vessel remains intact [112]. Another frequently used model is aortic arch banding (AAB) because it results in a progressive increase in left ventricular pressure, being more advantageous and effective than abdominal banding [108]. Published work by Schnelle et al. described a significant reduction in systolic and diastolic function in the hypertrophic model six weeks after AAB. Echocardiographic analysis described a reduction in EF and GLS, altered ventricular relaxation with increased E/e′ ratio, and increased LA size. In addition, there was an increase in the left ventricular weight to body weight ratio when comparing the treatment and control groups. This finding was also confirmed upon echocardiographic evaluation, which described eccentric left ventricular hypertrophy and increased end-diastolic dimensions of the LV [34].

Banding of the ascending portion of the aorta results in a high and rapid pressure increase in the left ventricle, which results in important ventricular hypertrophy within 48 h after surgery [113].

Another pattern of acute hypertrophy is determined by treatment with thapsigargin, a tumor promoter that increases the cytosolic calcium concentration by preventing the ion from entering sarcoplasmic or endoplasmic reticulum [114]. The acute model is associated with increased systolic impairment with a significant reduction in EF and GLS in response to elevated left ventricular pressures. Nevertheless, the surgical model remains more commonly used. Bauer and colleagues described an increase in left ventricular thicknesses and mass within 1 week after surgery and a reduction in GLS due to dyssynchrony and myocardial dysfunction from pressure overload [115]. They subsequently randomized some animals treated with banding of the ascending aorta to suture removal surgery, demonstrating a slight restoration of contractile function and substantial improvement in hypertrophy [115].

Furihata and colleagues evaluated pattern variability in response to the degree of constriction by describing a pattern of hypertrophy without decompensation (for less tight ligatures and a 0.400 mm caliber) and a pattern of hypertrophy with transition to HF in 4 weeks (for ligatures with a diameter of 0.385 mm). Significant hypertrophy of the LV was determined in the first group, while in the second group, there was also an increase in end-diastolic and end-systolic diameters of the ventricle and a decrease in FS. Tighter ligatures were associated with a lower survival rate for the onset of acute HF due to acute pressor overload [116,117]. The second model was described in a study by Patt and colleagues, who described a compensatory and a decompensatory phase. The first phase described biventricular concentric remodeling associated with hypertension. The second phase was associated with the development of HF with increased ventricular thicknesses, sizes, and volumes and reduced EF and FS [118].

Another surgical model involves the creation of the aortocaval fistula. The fistula is created by laparotomy; the suprarenal aorta and the vena cava are clamped, and then puncture of the aorta into the vena cava is performed. Successful surgery is confirmed by pulsatile distension of the vena cava and postoperative CFM. The creation of the shunt results in pressure overload. Aortocaval fistula causes cardiac hypertrophy and diastolic dysfunction, represented by an increase in the size of the LA. Regarding systolic function, mice exhibit a hyperkinetic ventricle with increased EF, FS, and GLS values [119].

Non-surgical models have been tested by using deoxycorticosterone acetate (DOCA) infusion and angiotensin II (ANG II). A model of HFpEF has been described in DOCA-salt mice: treatment with DOCA causes renal imbalance with increased sodium and water reabsorption, resulting in volume overload and hypertension. The pattern is usually complemented by a high-salt diet and often associated with unilateral nephrectomy with the chronic development of hypertension [120]. After two weeks, these mice develop reduced e′ and reduced E/e′ and e′/a′ ratios and thus reduced diastolic relaxation, moderate ventricular hypertrophy, and enlargement of the LA area without changes in systolic function [121].

Other model of hypertension has been developed in mice via chronic treatment with ANG II. Angiotensin binds to two specific receptors: receptor type 1 and receptor type 2. Angiotensin binding to AT1 results in vasoconstriction, the stimulation of aldosterone release, the promotion of cell growth, matrix deposition, and inflammation. This causes vascular remodeling characterized by medial thickening, perivascular fibrosis, inflammatory cell accumulation, oxidative stress, and subsequently hypertrophic cardiomyopathy [122,123]. Notably, ANG II infusion does not alter systolic function, whereas it results in increased parietal thicknesses and altered diastole with a reduced E peak and E/A ratio [124]. A Japanese study described four murine models of hypertension: model AT (infusion of ANG II), model AN (infusion of ANG II and uninephrectomy), model AS (infusion of ANG II and salt loading), and model ANS (the combination of ANG II, uninephrectomy, and salt loading) [125]. At 6 weeks, increased parietal thickness was described in all models. Furthermore, while in the AT, AN, and AS models there were no alterations in FS, the ANS model showed systolic dysfunction and congestive HF with increased mortality of 35.3% compared to the other groups because, at 5 weeks, there was an increase in left ventricular end-diastolic pressure, resulting in increased LVDd, reduced FS, and reduced systolic and diastolic output.

Chronic treatment with ISO also leads to increased ventricular mass and the development of a hypertrophic cardiomyopathy model [126]. Specifically, the chronic administration of ISO via subcutaneous mini-pumps induces cardiac hypertrophy during the first week [127]. During this compensatory stage, echocardiographic evaluation reveals an increase in IVSd, PWD, and FS [128]. In the following three weeks, the model evolves into advanced heart failure, characterized by a progressive increase in LVIDd and LVM, wall thinning, ventricular dilation, and reduced systolic function.

Moreover, the chronic ISO infusion model has been associated with increased susceptibility to atrial fibrillation and ventricular arrhythmias, which correlates with a higher incidence of sudden cardiac death in subjects with hypertrophic cardiomyopathy [67,128,129,130].

Hypertrophic cardiomyopathy (HCM) is hereditary in about half of diagnosed cases in humans and is associated with mutations in genes encoding sarcomeric proteins—most commonly myosin genes, which are involved in 40–60% of cases. Other causes may be genetic or non-genetic (5–10%), while in 25–30% of cases, the etiology remains undetermined [129]. New therapeutic strategies for the treatment of HCM may be beneficial for improving symptoms, enhancing quality of life, and preventing sudden cardiac death. Finally, a model of familial hypertrophic cardiomyopathy was generated in mice by the mutation of a sarcomeric protein. Harris and colleagues caused a deletion of myosin-binding protein C (MyBP-c), a component of sarcomere thick filaments, via a deletion of exons 3 and 6 from the gene. Mice with homozygosity mutation had a markedly hypertrophic and dilated phenotype with reduced EF and FS and impaired diastole [131].

**Table 5:** Murine models of pressure overload/hypertensive heart disease: mechanisms, characteristics, and echocardiographic assessment.

Mouse Model	Mechanism	Model	Main Features	Echocardiographic Assessment
Pressure Overload/Hypertensive heart disease	Surgical method	Ascending aortic constriction (AAC) [105]	LV pressure overload induced by aortic constriction	Eccentric cardiac hypertrophy + reduced sistolic function + dyastolic disfunction: - Increased IVSd/s and LVPWd/s - Reduced EF, FS, and GLS - Increased E/e′ ratio, LA area, and IVRT
		Transverse aortic constriction (TAC) [106]		
		Suprarenal abdominal aortic banding (AAB) [112]		
		Aortocaval fistula (shunt) [119]	LV volume overload induced by the creation of a shunt between the aorta and vena cava inferior	Eccentric cardiac hypertrophy + hypercontractile stage with increased sistolic function + dyastolic disfunction: - Increased IVSd/s and LVPWd/s - Increased EF, FS, and GLS - Increased LA area - Reduced IVRT
	Chemical induction	DOCA-salt + unilateral nephrectomy + 1% NaCl drinking water solution [120]	Renal imbalance with increased reabsorption of sodium and water resulting in hypervolemia	Concentric cardiac hypertrophy + preserved sistolic function + early dyastolic disfunction: - Increased IVSd/s and LVPWd/s - Preserved EF, FS, and GLS - Increased LA area - Reduced e′ wave velocity peak and inverted e′/a′ ratio - Increased E/e′ ratio
		Angiotensin II infusion (1,4 mg/kg/die) [122]	Increase blood pressure via vasoconstriction	Eccentric cardiac hypertrophy + hypercontractile stage with increased sistolic function + dyastolic disfunction: - Increased IVSs/d, LVPWs/d, and LVIDs/d - Increased EF, FS, and GLS - Reduced E wave velocity peak and E/A ratio - Increased E/e′ratio and IVRT
		Isoproterenol (30 mg/kg/die) [127]	Hypertrophic response to adrenergic stimulation: - Uniform hypertrophic response - Collagen increase - Increase in cardiac mass - Cardiotoxicity - Electrical remodeling	Concentric cardiac hypertrophy + hypercontractile stage with increased sistolic function + dyastolic disfunction: - Increased IVSs/d, LVPWs/d, and LVIDs/d - Increased EF, FS, and GLS - Reduced E wave velocity peak
	Genetically induced	Mybpc3−/−mice [131]	Mutation in endogenous cardiac (c) *MyBP-C* gene and absence of cMyBP-C results in familial hypertrophic cardiomyopathy	Eccentric cardiac hypertrophy + hypercontractile stage with increased sistolic function + dyastolic disfunction: - Increased IVSs/d, LVPWs/d, and LVIDs/d - Reduced EF and FS - Increased IVRT

Note: This table provides an overview of commonly used mouse models to study hypertensive heart disease. The mechanism of model induction (surgically, chemically, or genetically induced), the pathophysiology, and the main expected echocardiographic alterations are summarized. EF, ejection fraction; FS, fractional shortening; GLS, global longitudinal strain; IVRT, isovolumic relaxation time; IVSs/d, interventricular septum, diastole/systole; LA, left atrium; LVIDs/d, left ventricle internal diameter, diastole/systole; LVPWs/d, left ventricular posterior wall, diastole/systole; LVIDs/d, left ventricle internal diameter, diastole/systole.

### 3.4. Echocardiographic Assessment of Ischemic Heart Disease Murine Models

MI is among the leading causes of death worldwide [132,133]. Ischemic cardiomyopathy occurs as a result of coronary ischemia, which causes reduction or absence of coronary blood flow with electrical, metabolic, structural, and functional consequences at the myocardial level [134]. Murine models of ischemic heart disease are extensively used to study pathogenetic mechanisms and evaluate new therapies (Table 6).

After myocardial infarction, uneven left ventricular remodeling occurs. Therefore, the use of the M-mode in this model is incomplete. Much more representative are the 2-D echo images acquired in different planes and the calculation of EF using the Simpson method, which allows the calculation of the volume of blood ejected at each cycle on a volume basis [16,134]. Recently, 4-D echo has been shown to be more reliable than the M-mode and 2-D echo in heart volumetric evaluation, especially after MI [30]. Rutledge et al. showed that semi-automated 4-D echo provides rapid and precise volumetric measurements comparable to CMR values [135]. In addition, the function can be assessed by calculating the number of segments found to be normokinetic. Accordingly, the wall motility score index (WMSI) is calculated: the ventricle is divided into 10–16 segments, and each segment is assigned a score from 1 to 5 (normal, hypokinetic, akinetic, dyskinetic, or aneurysmal, respectively); the result is the sum of the scores divided by the number of segments analyzed [16].

Commonly used models are surgical models, divided into irreversible ischemia models (coronary artery ligation or ablation) and reversible ischemia/reperfusion (I/R) models. Such a model is commonly used for pioneering studies of cardiac regeneration [136]. However, these methods are associated with low rates of postoperative survival, bleeding, and pneumothorax [137]. Permanent MI induced by ablation methods is still uncommon and is achieved by electrocautery injury or cryogenic damage [138,139].

Permanent surgical ligation of the coronary artery requires anesthetizing and intubating the mice, performing a thoracotomy to expose the left ventricle, and tying the artery with a nylon suture. The left anterior descending (LAD) artery appears as a bright red line running from the edge of the LA to the apex of the heart. Bleaching of the ventricle downstream of the suture is an indication of successful ligation (wave of injury) [140]. Limitations to the procedure are due to the ligated artery tract and additionally to variability among animals [141]. As early as one week after the procedure, the ventricle appeared remodeled with an increased ventricular diameter [142]. Then, 28 days after LAD ligation, defective cardiac wall motion, increased vital ventricular mass and apical thinning (infarcted region), reduced EF, and the development of HF can be observed on an echocardiogram [143]. Strain assessment after IM proves to be very useful in describing adverse remodeling and ventricular dyssynchrony. In particular, several studies have described a reduction in GLS, GRS, and GCS of 11%, 12%, and 13%, respectively, after infarction in mice [144,145].

The technique for reversible ischemia is more recent and is performed using surgery. The LAD is temporarily ligated for a time ranging from 15 min to 2 h, depending on the protocol (optimal time 30 min) [146]. This technique has been tested to describe reperfusion injury: available oxygen after reperfusion results in the formation of reactive oxygen species (ROS); high levels of ROS activate chemokines, alter the function of mitochondria, and result in cell apoptosis that worsens necrosis in infarcted cardiac tissue (second wave of injury) [147,148]. Yang and colleagues described two murine I/R models of echocardiographic evaluation at baseline and 1 day, 3 days, and 42 days post-surgery. All animals showed a decrease in EF after 1 day. At day 3, however, two groups could be distinguished: the I/R group and failed I/R group. Animals in the I/R group had a 3-day EF < 45% and maintained a low EF at 42 days; in addition, the GLS and GRS of the anterior wall were altered. In contrast, the failed I/R group had an improved EF at day 3 (>45%) and progressive improvement in GLS and anterior wall GRS [24]. A closed-chest I/R model was described in studies by Dewald and Nossuli [149,150]. This model in Christia’s study was used for echocardiographic evaluation after reperfusion. Mice showed ventricular remodeling with dilatation of the infarcted heart chamber after 7 days and worsening of dilatation at 28 days. Non-infarcted segments showed progressive hypertrophy [151].

**Table 6:** Murine models of ischemic cardiomyopathy: mechanisms, characteristics, and echocardiographic assessment.

Mouse Model	Mechanism	Model	Main Features	Echocardiographic Assessment
Ischemic cardiomyopathy	Irreversible surgical methods	Permanent left anterior descending artery ligation [143]	Suture of the left anterior descending coronary artery with a needle; confirm the color change of the left ventricle within 10 s and ST elevation with an EKG monitor: - Irreversible hypoxia - Cell swelling, rupture, and death - Infarct scar formation - Remodeling	Ventricular dilation + reduced sistolic function + dyastolic disfunction: - Increased WMSI - Increased LVIDs/d, IVSd/s, and LVPWd/s - Reduced EF, FS, GLS, GCS, and GRS - Decreased e′ wave velocity peak - Increased * E/e′ ratio * Reperfusion results in less cardiac remodeling than permanent ischemia; in the IR model, there is an improvement in cardiac performance.
	Reversible surgical methods	Ischemia/reperfusion (I/R) model [151]	Temporary ischemia followed by restoration of blood flow: - Reversible or partially reversible hypoxia - Oxygen reintroduction attenuating cell swelling, rupture, and death - Oxygen can exacerbate injury by ROS, Ca^2+^, and inflammatory response - Smaller infarct scar and less extensive remodeling proportionate to duration of ischemia	Ventricular dilation + reduced sistolic function + dyastolic disfunction: - Increased * LVIDs/d, IVSd/s, and LVPWd/s - Reduced * EF, FS, GLS, GCS, and GRS - Decreased e′ wave velocity peak - Increased * E/e′ ratio * Reperfusion results in less cardiac remodeling than permanent ischemia; in the IR model, there is an improvement in cardiac performance.

Note: This table summarizes the main types of mouse models used to study ischemic cardiomyopathy. The mechanism of model induction (irreversible or reversible surgical method), the pathophysiology, and the main expected echocardiographic alterations are summarized. EF, ejection fraction; FS, fractional shortening; GLS, global longitudinal strain; GCS, global circumferential strain; GRS, global radial strain; IVRT, isovolumic relaxation time; IVSd/s, interventricular septum, diastole/systole; LVIDs/d, left ventricle internal diameter, diastole/systole; LVPWs/d, left ventricular posterior wall, diastole/systole. LVIDs/d, left ventricle internal diameter, diastole/systole, WMSI, wall motion score index.

## 4. Conclusions

Preclinical research in the cardiovascular field, along with the use of echocardiographic techniques applied to injury models, aims to replicate the pathology in animals in order to define the underlying mechanisms of damage and identify new therapeutic targets to improve prognosis or alleviate symptoms [152]. For this reason, echocardiography has become an indispensable tool used for the non-invasive assessment of cardiac structure and function in murine models of heart diseases. Its high temporal resolution, portability, cost-effectiveness, and ability to acquire serial measurements make it particularly well suited for preclinical cardiovascular research [33,153]. The technique allows for the simultaneous evaluation of multiple physiological parameters, including chamber dimensions, wall motion, systolic and diastolic function, valvular integrity, and pericardial involvement. Furthermore, the integration of advanced imaging modalities such as TDI and STE enables detailed myocardial functional analysis with high sensitivity, even in subtle or early-stage pathological changes.

Despite its numerous advantages, murine echocardiography for the evaluation of pathological models presents several technical and methodological limitations (Table 7). First, disease models themselves can yield divergent phenotypes: for example, dilated cardiomyopathy induced by genetic mutations shows marked inter-strain differences in ventricular dilation and contractile reserve between C57BL/6 and FVB mice, whereas pressure-overload models such as transverse aortic constriction display outcome sensitivity to small variations in band tightness and surgical technique [107,154]. Second, echocardiographic measurements exhibit significant operator and equipment dependencies: the ejection fraction and fractional shortening can vary by ±8–12% between observers and by up to 15% across platforms with different transducer frequencies or frame rates [6]. To mitigate these issues, consensus protocols—detailing image-quality criteria, standardized anesthetic regimens, gating practices, and reporting checklists—have been proposed, yet adoption remains inconsistent [6,16,134,155]. Finally, translational gaps between murine and human cardiac imaging arise from fundamental physiological differences—mice beat at 500–600 bpm versus humans at 60–100 bpm, and murine myocardial fiber architecture and chamber geometry differ substantially, limiting the direct extrapolation of strain or diastolic indices [156]. Emerging strategies such as humanized mouse models and computational scaling laws offer promising avenues to bridge these gaps, but further validation is needed. Collectively, these considerations underscore the importance of critical model-specific validation, rigorous standardization, and cautious interpretation when translating murine echocardiographic findings to human cardiac disease.

It is not always possible to directly translate preclinical results into clinical practice or human studies [88]. Many preclinical findings fail to transition into clinical research, a phenomenon with multifactorial causes. From an efficacy standpoint, cause–effect or treatment–benefit results observed in preclinical studies are based on models that often do not fully replicate the human pathological condition and fail to account for comorbidities.

Similarly, safety data obtained from animal models may not adequately reflect systemic toxicity, effects on target organs, or interactions under comorbid conditions. Validity and standardization issues are also linked to the absence of well-defined protocols, which are essential to ensure the reproducibility of experimental procedures. Collaboration between research centers and the development of clear, consensus-based guidelines for model utilization during the experimental phase are critical [159].

Variability associated with operator expertise, probe positioning, image acquisition quality, the influence of anesthesia on cardiac function, and subsequent measurement of parameters significantly impacts the reliability of results obtained from murine models of cardiac injury [157,158].

Inter-operator variability represents a major limitation to reproducibility. This variability is often due to the lack of standardized training programs for operators, who typically rely more on practical experience than on formal protocol-based instruction [3]. Updating existing guidelines and developing validated documentation for image acquisition, as well as the definition of normal and pathological reference values, would provide strong support for the standardization of results [160].

The successful implementation of murine echocardiography demands rigorous standardization of acquisition protocols, dedicated training of personnel, and attention to technical detail [3]. Continuous waveform monitoring, image optimization, and validation of derived parameters are critical steps to ensure data reliability and reproducibility. Hereafter, a concise checklist is provided to improve acquisition protocols and reproducibility across laboratories:

- Imaging Settings: 30–40 MHz probe; ≥200 fps for 2D; ≥1 kHz sampling for PW Doppler; narrow sector width; focus at mid-myocardium.
- Anesthesia and Monitoring: Isoflurane 1–1.5% with a 10 min stabilization period; maintain heart rate at 400–600 bpm; keep body temperature at 37 ± 0.5 °C.
- Gating and Acquisition: ECG and respiratory gating; optimize gain for clear endocardial borders; acquire ≥ 3 stable cine-loops per view.
- Quality Control and Reporting: Operator training with blinded re-reads (CV < 10%); annual equipment calibration; report imaging view, probe settings, heart rate, anesthetic protocol, gating method, and refer to the international consensus for reference values.

Future directions in preclinical imaging include the broader adoption of 3-D echo, which provides more comprehensive volumetric data, and the enhanced use of STE-based strain imaging, which is increasingly recognized for its superior sensitivity in detecting myocardial injury compared to conventional metrics like EF or FS. The integration of advanced techniques promises to transform the field. AI-driven platforms, deep convolutional neural networks, enable fully automated, observer-independent quantification of chamber volumes, ejection fraction, and strain with expert-level precision [161]. These innovations in AI will enable unprecedented sensitivity, reproducibility, and throughput, hastening the translation of murine findings into human cardiac applications.

In conclusion, when applied with appropriate technical rigor and physiological consideration, echocardiography represents a robust and versatile modality for the assessment of cardiac function in mice, with significant potential to advance cardiovascular research and bridge the gap between bench and bedside.

## Figures and Tables

**Table 1:** Reference echocardiographic parameters in healthy adult C57BL/6 mice.

Echocardiographic Parameters		Adult C57BL/6
Morphology [6]	IVSd (mm)	0.71 ± 0.15
	LVIDd (mm)	3.69 ± 0.41
	LVPWd (mm)	0.79 ± 0.22
	IVSs (mm)	0.97 ± 0.19
	LVIDs (mm)	2.20 ± 0.50
	LVPWs (mm)	1.12 ± 0.33
Systolic function [6]	EF (%)	71 ± 11
	FS (%)	43 ± 9
Diastolic function [6]	E (mm/s)	718 ± 109
	A (mm/s)	455 ± 105
	E slope (mm/s)	19.87 ± 1.67
	E/A	1.52 ± 0.40
	e′ (mm/s)	43.2 ± 10.9
	E/e′	15.2 ± 6.7
Strain [7]	GLS (%)	−22
	GRS (%)	35
	GCS (%)	−30

Note: A, late diastolic transmitral flow velocities; E, early diastolic transmitral flow velocities; e′, peak early diastolic annular velocity; EF, ejection fraction; FS, fractional shortening; IVSd, interventricular septum thickness in diastole; IVSs, interventricular septum thickness in systole; LVIDd, left ventricle internal diameter in diastole; LVIDs, left ventricle internal diameter in systole; LVPWd, left ventricular posterior wall thickness in diastole; LVPWs, left ventricular posterior wall thickness in systole; GCS, global longitudinal strain; GLS, global circumferential strain; GRS, global radial strain.

**Table 2:** How to perform a comprehensive sequential segmental TTE in mouse. This table serves as a practical tool for standardizing echocardiographic acquisitions in animal models of heart disease. It describes the three main echocardiographic views, parasternal long-axis (PLAX), parasternal short-axis (PSAX), and apical four-chamber (A4C), used to perform a comprehensive transthoracic echocardiography in mice. For each imaging window, the position of the transducer and the animal, the echocardiographic modalities employed, the anatomical structures visualized, and the morphological and functional parameters that can be assessed are described.

View	Modality	Anatomy	Measurement ± Explanayory Note
PLAX [16] - Left side of the animal’s chest - Probe marker toward the left hip - Platform, supine position	2D [2]	LV outflow tract, MV Ao, LV, and LA	LV assessment: - Antero-septum wall - Posterior wall
	MM [13]	LV	Morphology: - LVIVS d/s - LVPWD d/s - LVID d/s - LVEDV d/s Systolic function: - EF - FS
	STE [17]	LV	GLS: - Posterior wall: basal, mid, apex - Antero-septum wall: basal, mid, apex
PSAX [16] - Left side of the animal’s chest - Probe marker ~90° clockwise of the PLAX - Platform, supine position	2D [2]	LV and RV	LV assessment: - Anterior free wall and septum - Lateral wall - Posterior wall - Inferior free wall
	MM [13]	LV	Morphology: - LVIVS d/s - LVPWD d/s - LVID d/s - LVEDV d/s Systolic function: - EF - FS
	STE [17]	LV	GRS: - Anterior free wall - Lateral wall - Posterior wall - Inferior free wall - Anterior septum
	STE [17]	LV	GCS: - Anterior free wall - Lateral wall - Posterior wall - Inferior free wall - Anterior septum
A4C [16] - Apical - Probe marker ~7–8 o’clock, - Transducer tilted by ~30–60° relative to the platform - Platform, leftward trendelenburg position (25–30°)	2D [2] CFM [18]	Full heart sweep (LA, LV, MV, RA, RV, TV)	Full heart visualization: - LA size - Left atrioventricular valve appearance - Ventricular morphology, size, thickness, and function
	PWD [19]	MV	Diastolic function: - E velocity - A velocity - E deceleration time
	TDI [20]	MV	Diastolic function: - e′ velocity - a′ velocity

Table Note: A, late diastolic transmitral flow velocities; A4C, apical four chamber; Ao, aorta; CFM, color flow mapping; E, early diastolic transmitral flow velocities; e′, peak early diastolic annular velocity; EF, ejection fraction; FS, fractional shortening; GCS, global longitudinal strain; GLS, global circumferential strain; GRS, global radial strain; LA, left atrium; LV, left ventricle; LVIVS d/s, left ventricle interventricular septum thickness, diastole/systole; LVID d/s: left ventricle internal diameter, diastole/systole; LVPWD d/s: left ventricle posterior wall diameter, diastole/systole; MM, M-mode; MV: mitral valve; PLAX: parasternal long axis; PSAX: parasternal short axis; PWD: pulsed wave Doppler; RA: right atrium; RV: right ventricle; STE: speckle-tracking echocardiography; TDI: tissue Doppler imaging; TV, tricuspid valve.

**Table 3:** Murine models of non-ischemic dilated cardiomyopathy: mechanisms, characteristics, and echocardiographic assessment.

Mouse Model	Mechanism	Model	Main Features	Echocardiographic Assessment
Non-ischemic dilated cardiomyopathy	Drug-induced toxic cardiomyopathy	Single high-dose doxorubicin (15–20 mg/kg) [50]	Acute cardiotoxic action (5 days) through the formation of reactive oxygen species and mitochondrial damage by DNA breakage due to the doxorubicin–topoisomerase 2β complex: - Reduced activity - Increased BNP - Cytoplasmic vacuolation - Myofibrillar disarray - Swelling of the sarcoplasmic reticulum - Increased myocardial cell necrosis	Acute dilated cardiomyopathy + reduced sistolic function + diastolic dysfunction: - Increased LVIDs/d - Reduced IVSd/s and LVPWs/d - Reduced EF, FS, GLS, GCS, and GRS - Reduced E and e′ waves’ velocity peaks
		Multiple low-dose doxorubicin (4–5 mg/kg for 5 weeks) [51]	Chronic cardiotoxic action (4–8 weeks) through the formation of reactive oxygen species and mitochondrial damage by DNA breakage due to the doxorubicin–topoisomerase 2β complex: - Myocardial cell edema and vacuolar degeneration - Myocardial fiber rupture - Cardiomyocytes’ small focal or patellar necrosis	Chronic dilated cardiomyopathy + reduced sistolic function + dyastolic disfunction: - Increased LVIDs/d - Reduced IVSd/s and LVPWs/d - Reduced EF, FS, GLS, GCS, and GRS - Reduced E wave velocity peak - Increased E/e′ ratio - Prolonged IVRT
		Isoproterenol(400 mg/kg) [52]	Stress-induced cardiomyopathy due to excessive adrenergic stimulation: - ER stress - Apoptosis - Downregulation of beta-adrenergic receptors and adenyl cyclase activities	Acute and reversible dilated cardiomyopathy + reduced sistolic function + dyastolic disfunction: - Increased LVIDs/d - Reduced IVSd/s and LVPWs/d - Reduced EF, FS, GLS, and GCS - Reduced E and e′ waves’ velocity peaks
		Isoproterenol(200 mg/kg) + 5-fluorouracile (15 mg/kg/day) [53]	Dilated cardiomyopathy resulting from acute Isoproterenol exposure followed by 5-fluorouracil administration (anti-mitotic agent 5-F): - Persistent cardiomyocyte damage - Premature cell senescence cardiomyocyte death due to necrosis and apoptosis - Chronic myocardial inflammatory reaction ROS increase - Fibrosis (scar tissue)	Chronic dilated cardiomyopathy + reduced sistolic function: - Increased LVIDs/d - Reduced IVSd/s and LVPWs/d - Reduced EF, FS, and GLS - Reduced E and e′ waves’ velocity peaks - Increased E/e′ ratio
		Ethanol [54]	Direct cardiotoxicity of ethanol and its metabolites, oxidative stress, and accumulation of fatty acid ethyl esters: - Disruption of myofibrillary architecture - Myocardial dysfunction	Chronic dilated cardiomyopathy + reduced sistolic function: - Increased LVIDs/d - Reduced IVSd/s and LVPWs/d - Reduced EF and FS
		Homocistein [55]	Chelates copper and impairs copper-dependent enzymes: - Fibrosis - Endothelial dysfunction	Chronic dilated cardiomyopathy + left ventricular hypertrophy + reduced sistolic function: - Increased LVIDs/d, IVSd/s, and LVPWs/d - Reduced EF and FS
	Genetically induced	D230NcTm [56]	Reduced Ca^2+^ sensitivity of troponin C in the sarcomere, resulting in reduced cardiac contractility	Dilated cardiomyopathy + reduced sistolic function + dyastolic disfunction: - Increased LVIDs/d - Reduced IVSd/s and LVPWd/s - Reduced EF and FS
		D73NcTnC [57]	Reduced Ca^2+^ sensitivity of troponin C in the sarcomere, resulting in reduced cardiac contractility	
		I61QcTnC [58]	Reduced Ca^2+^ sensitivity of troponin C in the sarcomere, resulting in reduced cardiac contractility	

Table Note: This table summarizes the main mouse models used to study non-ischemic dilated cardiomyopathy. The mechanism of model induction (drug- or genetically induced), the pathophysiology, and the main expected echocardiographic alterations are summarized. EF, ejection fraction; FS, fractional shortening; IVSd/s, interventricular septum, diastole/systole; LVIDs/d, left ventricle internal diameter, diastole/systole; LVPWs/d, left ventricular posterior wall, diastole/systole.

**Table 4:** Murine models of diabetic cardiomyopathy (T1D and T2D): mechanisms, characteristics, and echocardiographic assessment.

Mouse Model	Mechanism	Model	Main Features	Echocardiographic Assessment
Diabetic Cardiomyopathy (T1D)	Spontaneous autoimmune	NOD mice [82]	Infiltration and destruction of β cells by T cells (CD4 and CD8), NK cells, and B cells (insulitis): - Hyperinsulinemia - Hyperglicemia - Polyuria - Polydipsia	Preserved sistolic function + early dyastolic disfunction: - Preserved EF, FS, and LVID s/d - Small reduction in GLS - Reduced E and e′ waves’ velocity peaks - Increased E/e′ ratio
	Genetically induced	AKITA mice [83]	Mutation in insulin 2 gene (Ins2/Cys96Tyr) and overload of misfolded insulin (ER stess): - Hyperinsulinemia - Hyperglicemia - Polyuria - Polydipsia	
	Chemical induction	High-dose streptozocin (100–200 mg/kg) [84]	Rapid ablation of β cells (DNA damage): - Hyperglicemia	Ventricular dilation ± dyastolic disfunction ± sistolic disfunction: - Increased LVID s/d - Reduced EF, FS, and GLS - Reduced E and e′ waves’ velocity peaks - Prolonged E slope - Increased E/e′ ratio
		Alloxan (50–200 mg/kg) [85]	Infiltration of β cells and formation of free radicals (redox cicle): - Hyperglicemia	
Diabetic Cardiomyopathy (T2D)	Chemical induction	Multiple low-dose streptozocin (20–40 mg/kg for 5 days) [84]	Infiltration of β cells by macrophages and T and B cells and reduction in islet numbers: - Hyperglicemia	Preserved sistolic function + early dyastolic disfunction: - Preserved EF, FS, and LVID s/d - Small reduction in GLS - Reduced E and e′ waves’ velocity peaks - Prolonged E slope - Increased E/e′ ratio
	Induced obesity	High-fat diet [86]	Normal chow exchanged with a high-fat diet (58% fat): - Hyperinsulinemia - Insulin resistance - Impaired glucose tollerance obesity	Left ventricular hypertrophy + preserved sistolic function + early dyastolic disfunction: - Increased IVS s/d, LVPW s/d, and LVID s/d - Preserved EF and FS - Reduced e′ wave velocity peak - increased E/e′ and E/A ratio
	Obese models	Lep^ob/ob^ mice [87]	Deficient in leptin: - Hyperglicemia - Hyperinsulinemia - Hyperlipidemia - Obesity	Left ventricular hypertrophy + preserved sistolic function + early dyastolic disfunction: - Increased IVSs/d and LVPWs/d - Preserved EF, FS, and LVIDs/d - Reduced e′ wave velocity peak - Increased a′ wave velocity - Inverted e′/a′ ratio - Increased E/e′ and E/A ratio
		Lepr^db/db^ mice [88]	Autosomal recessive mutation in the leptin receptor: - Hyperglicemia - Hyperinsulinemia - Hyperlipidemia - Obesity	

Table Note: This table summarizes the main mouse models used to study type 1 and type 2 diabetic cardiomyopathy (T1D and T2D). The mechanism of model induction (spontaneous, genetic, or chemical induction), the pathophysiology, and the main expected echocardiographic alterations are summarized. EF, ejection fraction; FS, fractional shortening; GLS, global longitudinal strain; IVS s/d, interventricular septum, diastole/systole; LVID s/d, left ventricle internal diameter, diastole/systole; LVPW s/d, left ventricular posterior wall, diastole/systole.

**Table 7:** Advantages and limitations of echocardiography in murine models.

Technique	Advantages	Disadvantages
Echocardiography [155,157,158]	Widely available Portable Cost-effective Suitable for individual use in laboratories Fast measurements (with experience) Enables serial assessments Simultaneous measurement of a broad range of physiological parameters Enables evaluation of chambers, pericardium, valves, deformation, and function High temporal resolution Can be performed in awake or anesthetized animals - Awake: no anesthetic effects - Anesthetized: easier handling and probe positioning	Technical variability (probe position and image acquisition line) if the operator is not highly trained Requires quality control of image acquisition and data analysis Animal acclimatization needed, especially for serial measurements in the same mouse Awake: may induce stress; heart rates <650 bpm often reflects stress conditions - Requires enrichment and operator training Anesthetized: risk of over-/under-dosing; heart rate must be maintained between 400 and 650 bpm to ensure physiological relevance

## Data Availability

No new data were created for this review article.

## Abbreviations

The following abbreviations are used in this manuscript:

**Abbreviation**	**Full Term.**
2-D echo	Two-Dimensional Mode.
3-D echo	Three-Dimensional Echocardiography.
4-D echo	Four-Dimensional Echocardiography.
5-FU	5-Flurouracil.
A	A Wave.
A4C	Apical Four-Chamber.
AAB	Aortic Arch Banding.
AI	Artificial Intelligence.
ANG II	Angiotensin II.
AO	Aorta.
AV	Aortic Valve.
B-mode	Two-Dimensional Mode.
BW	Body Weight.
BPM	Beats Per Minute.
CFM	Color Flow Mapping.
CMR	Cardiac Magnetic Resonance.
CO	Cardiac Output.
CWD	Continuous-Wave Doppler.
DCM	Dilated Cardiomyopathy.
DOCA	Deoxycorticosterone Acetate.
DT	Deceleration Time.
E	E Wave.
ECG	Electrocardiographic.
EF	Ejection Fraction.
FS	Fractional Shortening.
GCS	Global Circumferential Strain.
GLS	Global Longitudinal Strain.
GRS	Global Radial Strain.
HF	Heart Failure.
HFD	High-Fat Diet.
HFpEF	Heart Failure With Preserved Ejection Fraction.
HFrEF	Heart Failure With Reduced Ejection Fraction.
I/R	Ischemia/Reperfusion.
ISO	Isoproterenol.
IVRT	Isovolumic Relaxation Time.
LA	Left Atrium.
LAD	Left Anterior Descending.
LV	Left Ventricle.
LVEDV	Left Ventricular End-Diastolic Volume.
LVESV	Left Ventricular End-Systolic Volume.
LVIDd	Ventricular End-Diastolic Internal Diameter.
LVIDs	Left Ventricular End-Systolic Internal Diameter.
LVM	Left Ventricular Mass.
MI	Myocardial Infarction.
M-mode	Motion Mode.
MyBP-c	Myosin-Binding Protein C.
NOD	Non-Obese Diabetic.
PLAX	Parasternal Long-Axis.
PM	Papillary Muscle.
PSAX	Parasternal Short-Axis.
PWD	Pulsed-Wave Doppler.
ROI	Region Of Interest.
ROS	Reactive Oxygen Species.
RV	Right Ventricle.
SIC	Stress-Induced Cardiomyopathy.
STE	Speckle-Tracking Echocardiography.
STZ	Streptozotocin.
SV	Stroke Volume.
T1D	Type 1 Diabetes Mellitus.
T2D	Type 2 Diabetes Mellitus.
TAC	Transverse Aortic Constriction.
TDI	Tissue Doppler Imaging.
TTE	Transthoracic Echocardiography.
TTP	Time To Peak.
WMSI	Wall Motility Score Index.
